# The Role of Cytoreductive Nephrectomy and Targeted Therapy on Outcomes of Patients With Metastatic Sarcomatoid Renal Cell Carcinoma: A Population-Based Analysis

**DOI:** 10.7759/cureus.25395

**Published:** 2022-05-27

**Authors:** Taha Al-Juhaishi, Xiaoyan Deng, Dipankar Bandyopadhyay, Asit Paul

**Affiliations:** 1 Hematology and Medical Oncology, University of Oklahoma Health Sciences Center, Oklahoma City, USA; 2 Biostatistics, Virginia Commonwealth University, Richmond, USA; 3 Hematology and Medical Oncology, Virginia Commonwealth University School of Medicine, Richmond, USA

**Keywords:** nephrectomy, cancer outcomes, national cancer database and seer analyses, sarcomatoid renal cell carcinoma, renal cell carcinoma (rcc)

## Abstract

Sarcomatoid renal cell carcinoma (sRCC) is a rare but aggressive form of kidney cancer with a poor prognosis. Despite recent advances in therapies for kidney cancers, an effective management strategy for sRCC is uncertain. We evaluated the impact of targeted therapy and cytoreductive nephrectomy (CN) on survival outcomes of patients with metastatic sRCC. We identified patients diagnosed with sRCC between January 1, 1973, and December 31, 2014, within the Surveillance, Epidemiology and End Results (SEER) database. Patients with metastatic sRCC were stratified based on the era of diagnosis (before or after the introduction of targeted systemic therapy in 2006) and the status of CN. Cancer-specific survival (CSS) and overall survival (OS) were analyzed. Data of 993 patients with metastatic sRCC were available for analysis. The median age was 62 years. Most patients were male (69%), Caucasian (71%), and were diagnosed in the targeted therapy era (83%); 53% of patients underwent CN. CSS and OS of the whole cohort were 5.0 months and 4.0 months, respectively. While the introduction of targeted therapy did not improve outcomes, CN improved CSS and OS in both pre-targeted therapy and targeted therapy era. On multivariable analysis, CN was a predictor of an improved CSS (hazard ratio [HR] 0.54, p < 0.0001) and OS (HR 0.51, p* *< 0.0001). Among other factors, older age at diagnosis, higher T stages, and node positivity were associated with worse outcomes. Our results showed that the introduction of targeted therapy did not improve outcomes in patients with metastatic sRCC. CN improved survival in both pre-targeted and targeted therapy eras.

## Introduction

The American Cancer Society estimates 79,000 new cases and 13,920 deaths from cancers of the kidney and renal pelvis in 2022 in the US [1]. Renal cell carcinoma (RCC) is the most common type of kidney cancer. Sarcomatoid RCC (sRCC) is a rare form of RCC comprising about 5-20% of RCCs [2,3]. sRCC is not a distinct histologic subtype of RCC but is a de-differentiation of an epithelial neoplasm to a spindle cell phenotype [4,5]. Pure sRCC is rare, and sarcomatoid components are present in variable proportions in other histologic subtypes of RCC. Sarcomatoid transformation confers a poor prognosis [6]. The majority of patients with sRCC have a large primary tumor, metastatic disease at presentation, and a median survival time of fewer than 12 months, despite aggressive treatment [2,3,6]. The biology of sRCC is poorly understood, and its response to conventional therapy for RCC is disappointing. An optimal management strategy for patients with sRCC remains elusive.

Nephrectomy is considered a potentially curative option for patients with early-stage RCC. Prospective studies prior to the availability of the targeted agents and population-based studies in the targeted therapy era also showed survival benefits of nephrectomy in the presence of metastatic disease (known as cytoreductive nephrectomy [CN]) in patients with clear cell RCC [7,8]. Nevertheless, two recent prospective clinical trials, CARMENA and SURTIME, failed to show any benefit of CN in the targeted therapy era [9,10]. The role of CN in patients with metastatic RCC (mRCC) is, therefore, currently debatable. sRCC is more aggressive and biologically distinct than conventional clear cell RCC, and the results of these trials are unlikely to apply to patients with sRCC. Currently available published data of CN in patients with sRCC are exclusively based on retrospective analysis and show mixed results [11-15]. The optimal selection of patients with mRCC, including those with sRCC, who will benefit from CN continues to evolve.

Systemic therapy for patients with RCC has evolved over the last two decades [16]. In December 2005, the US Food and Drug Administration (FDA) approved the first targeted therapy, sorafenib, for mRCC. Several other anti-angiogenic tyrosine kinase inhibitors (TKIs), mammalian target of rapamycin (mTOR) inhibitors, and immune checkpoint inhibitors (ICIs) - alone or in combination with a TKI or CTLA4 inhibitor - have since been approved. Randomized clinical trials have shown improved survival using these agents in patients with mRCC. Nevertheless, an overwhelming majority of these trials only included patients whose disease had clear cell histology. Recent data have shown the benefits of ICI-based combination therapies in patients with metastatic sRCC [17-19]. This led to the recommendation of ICI-based combination therapy for metastatic sRCC by the Society of Immunotherapy of Cancer [20]. Recent National Cancer Center Network guidelines (NCCN Version 4.2021) do not include a specific systemic therapy recommendation for metastatic sRCC [21]. European Society for Medical Oncology (ESMO) provides ICI combination as a grade A recommendation and the targeted agent sunitinib as a grade B recommendation, based on retrospective studies [22].

In this study, data from the Surveillance, Epidemiology, and End Results (SEER) database spanning more than four decades were analyzed. SEER is considered a comprehensive source of cancer incidence and survival in the US. SEER currently collects and publishes cancer incidence and survival data from population-based cancer registries covering approximately 48.0% of the US population Specifically, the impact of CN on survival outcomes in patients with metastatic sRCC was examined. The survival of patients with metastatic sRCC was compared before and after the introduction of systemic targeted agents.

## Materials and methods

Study population and design

A retrospective analysis of patients with sRCC was conducted using the SEER database of the National Cancer Institute. Patients with primary sRCC (Code 8318/3) diagnosed between January 1, 1973, and December 31, 2014, were identified using SEERstat software. Since the SEER database provides de-identified patient data and is available for public use, no institutional review board approval was required for the conduct of this study. Among the patients identified, data of patients with metastatic sRCC (M1) were analyzed.

Patients were stratified based on the year of diagnosis and CN status. The targeted agent sunitinib was approved by the FDA to treat advanced RCC in 2006. This study considered 2006 as the beginning of the targeted therapy era, as was used in other prior studies [8,11]. The pre-targeted therapy era was defined as before 2006, and the targeted therapy era was defined as 2006 or later. Analyses were performed on data through the end of 2014.

In the pre-targeted therapy era, the systemic drugs available for RCC were interleukin-2 and interferon-alpha (with bevacizumab). During the targeted therapy era (2006-2014), targeted drugs that were FDA approved and available for use included anti-angiogenic drugs (sorafenib, sunitinib, bevacizumab, pazopanib, and axitinib) and mTOR inhibitors (temsirolimus and everolimus) [11,12,23-27]. Chemotherapy agents were available for sRCC in both eras [28-30]. Chemotherapy was also combined with the targeted agent sunitinib based on limited data [30].The staging classification was based on the tumor, node, metastasis (TNM) staging by AJCC, 6th edition, provided in the database.

Measures and outcomes

The primary outcomes were cancer-specific survival (CSS) and overall survival (OS). Survival was defined as the time from cancer diagnosis until death from any cause (OS) or until death attributable to their cancer (CSS). Patients who were alive at the time of the last follow-up were censored. Survival analyses were stratified based on the status of CN and era of diagnosis (pre-targeted therapy era and targeted therapy era). Other co-variables of interest were age at diagnosis, gender, race, and T and N stages associated with metastatic disease.

Statistical analyses

For patient baseline characteristics, Wilcoxon, chi-square, and Fisher's exact tests were used to determine whether the distribution of each characteristic was significantly different in the pre-targeted therapy era versus the target therapy era. Survival analyses were performed using the log-rank Test (LIFETEST procedure) or Cox proportional hazards model (PHREG procedure). SAS (Statistical Analysis System) software (v.9.4; SAS Institute, Cary, NC) was used in all analyses. Adjusted hazard ratios (HRs) and 95% confidence intervals, obtained from fitting a Cox proportional hazards model, were reported. The two-sided significance level was set to 5% for assessing the significance of the estimated parameters. Kaplan-Meier survival curves were generated by using R (v.4.05). For all analyses, a p-value <0.5 was considered statistically significant.

## Results

Patient characteristics

A total of 2,523 patients with sRCC were identified in the SEER database within the abundant time period of interest (1973-2014). Patients who had non-metastatic disease (n = 860) and indeterminate M stage (n = 670) were excluded. Data from 993 patients with metastatic sRCC were available for analysis.

Demographic data are presented in Table 1. Age, gender, race, and T and N stages were balanced in the pre-targeted and targeted therapy era groups. The majority of patients were diagnosed with sRCC in the targeted therapy era (83.3%) and were male (69.4%) and white (71.0%) with T3 (40.3%) and N0 disease (47.4%) and had undergone CN (52.9%). More patients underwent CN in the pre-targeted therapy era (61.5%) than in the targeted therapy era (51.2%).

**Table 1:** Baseline characteristics of patients Data  are expressed as count (%), except for age.

	Pre-targeted Therapy Era	Targeted Therapy Era	Total	p-Value		
					n=166	n = 827	n = 993			
	Age, median year (range)	62 (27.0-95.0)	62 (19.0-93.0)	62 (19.0-95.0)	0.7919		
Gender				0.9735		
Female	51 (30.72)	253 (30.59)	304 (30.61)			
Male	115 (69.28)	574 (69.41)	689 (69.39)			
Race				0.6656		
Non-Hispanic White	114 (68.67)	591 (71.46)	705 (71.00)			
Non-Hispanic Black	19 (11.45)	80 (9.67)	99 (9.97)			
Non-Hispanic American Indian/Alaska Native	1 (0.60)	8 (0.97)	9 (0.91)			
Non-Hispanic Asian or Pacific Islander	6 (3.61)	45 (5.44)	51 (5.14)			
Non-Hispanic Unknown Race	0 (0.00)	1 (0.12)	1 (0.10)			
Hispanic	26 (15.66)	102 (12.33)	128 (12.89)			
T Stage				0.0847		
T0	0 (0.00)	10 (1.21)	10 (1.01)			
T1	12 (7.23)	97 (11.73)	109 (10.98)			
T2	22 (13.25)	86 (10.40)	108 (10.88)			
T3	59 (35.54)	341 (41.23)	400 (40.28)			
T4	45 (27.11)	172 (20.80)	217 (21.85)			
TX	28 (16.87)	121 (14.63)	149 (15.01)			
N Stage				0.0587		
N0	75 (45.18)	396 (47.88)	471 (47.43)			
N1	33 (19.88)	193 (23.34)	226 (22.76)			
N2	22 (13.25)	126 (15.24)	148 (14.9)			
NX	36 (21.69)	112 (13.54)	148 (14.9)			
Nephrectomy				0.0153		
Yes	102 (61.45)	423 (51.15)	525 (52.87)			
No	64 (38.55)	404 (48.85)	468 (47.13)			

Targeted therapy, CN, and survival

**Figure 1:**
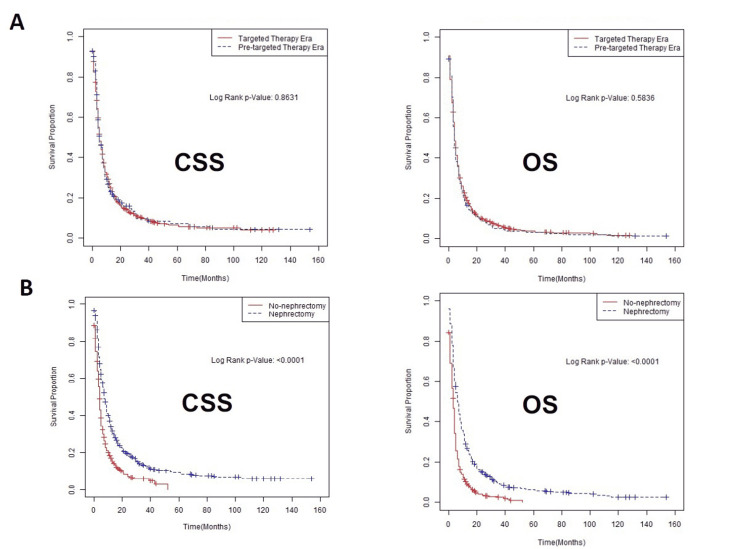
Kaplan-Meier survival curves showing CSS and OS of all patients with metastatic sRCC, stratified by eras of diagnosis (A) and status of cytoreductive nephrectomy (B). While both CSS and OS were not different between pre-targeted therapy and targeted therapy era, cytoreductive nephrectomy improved survivals. CSS, cancer-specific survival; OS, overall survival; sRCC, sarcomatoid renal cell carcinoma.

Overall, the CSS and OS of patients with mRCC continued to be poor at five months and four months, respectively, despite the introduction of targeted therapy. Kaplan-Meier survival curves for CSS and OS are shown in Figure 1. There were no differences in CSS and OS between the targeted therapy era and the pre-targeted therapy era. However, the CSS and OS of patients who underwent CN were better than those of patients who did not have CN. The comparative survival, stratified by the status of CN and era of diagnosis, is shown in Table 2. The survival benefits associated with CN were observed in both the pre-targeted therapy and targeted therapy eras.

**Table 2:** Cancer-specific and overall survival stratified by nephrectomy status and diagnosis era Data are expressed in median in months (95% confidence interval). CN, cytoreductive nephrectomy.

	Number of Subjects	No CN	CN	p-Value
Cancer-specific survival				
Pre-targeted therapy era	166	4.0 (3.0-5.0)	7.0 (4.0-9.0)	0.0162
Targeted therapy era	827	4.0 (3.0-4.0)	8.0 (7.0-9.0)	<0.0001
Overall survival				
Pre-targeted therapy era	166	4.0 (3.0-4.0)	5.0 (3.0-7.0)	0.0029
Targeted therapy era	827	3.0 (3.0-4.0)	7.0 (6.0-8.0)	<0.0001

Survival analyses with multiple covariates

The results of the Cox model survival analyses are summarized in Table 3. For CSS, older age at diagnosis, higher T stages (T3, T4), and lymph node positivity (N1, N2) were predictors of poor outcome. Older age at diagnosis, T4 stage, and node positivity (N1, N2) had worse OS. While the era of diagnosis did not predict survival, CN was a predictor of improved CSS (HR 0.54, p < 0.0001) and OS (HR 0.51, p < 0.0001).

**Table 3:** Hazard ratios from Cox model with multiple covariates CI, confidence interval.

Parameter (Reference)	Variable	Hazard Ratio	95% CI		p-Value
			Lower	Upper	
Cancer-Specific Survival					
Age at diagnosis (Unit = 1 year)		1.010	1.003	1.017	0.0027
Sex (Male)	Female	1.101	0.943	1.285	0.2216
Race (Hispanic)	Non-Hispanic American Indian/Alaska Native	0.732	0.339	1.583	0.428
	Non-Hispanic Asian or Pacific Islander	1.089	0.759	1.563	0.6429
	Non-Hispanic Black	1.291	0.96	1.735	0.0912
	Non-Hispanic Unknown Race	2.477	0.34	18.06	0.3708
	Non-Hispanic White	1.009	0.814	1.251	0.9362
Nephrectomy (No)	Yes	0.543	0.456	0.647	
Era (Nontargeted Era)	Targeted Era	0.988	0.814	1.198	0.8998
T Stage (T0)	T1	1.899	0.764	4.72	0.1674
	T2	2.292	0.921	5.704	0.0747
	T3	2.634	1.071	6.477	0.0349
	T4	2.823	1.146	6.953	0.0241
	TX	2.601	1.055	6.414	0.038
N Stage (N0)	N1	1.287	1.07	1.548	0.0074
	N2	1.214	0.981	1.501	0.0746
	NX	0.916	0.714	1.175	0.4884
Overall Survival					
Age at diagnosis (Unit = 1year)		1.019	1.013	1.025	
Sex (Male)	Female	1.032	0.895	1.189	0.6675
Race (Hispanic)	Non-Hispanic American Indian/Alaska Native	0.73	0.355	1.501	0.3925
	Non-Hispanic Asian or Pacific Islander	1.007	0.715	1.419	0.9668
	Non-Hispanic Black	1.306	0.994	1.717	0.0554
	Non-Hispanic Unknown Race	2.375	0.327	17.258	0.3926
	Non-Hispanic White	1.026	0.841	1.253	0.7985
Nephrectomy (No)	Yes	0.511	0.436	0.6	
Era (Nontargeted Era)	Targeted Era	0.914	0.769	1.085	0.3039
T Stage (T0)	T1	1.319	0.662	2.629	0.4312
	T2	1.53	0.765	3.062	0.2293
	T3	1.799	0.912	3.55	0.0902
	T4	1.993	1.009	3.934	0.047
	TX	1.82	0.922	3.595	0.0844
N Stage (N0)	N1	1.263	1.066	1.497	0.007
	N2	1.251	1.03	1.519	0.0241
	NX	0.922	0.739	1.149	0.4677

## Discussion

Despite advances in systemic therapies for RCC in recent years, patients with metastatic sRCC continue to have poor outcomes, and current management strategies are inadequate [2,3].

This study examined the impact of targeted therapy and CN on the outcomes of 993 patients with metastatic sRCC, spanning over four decades, in the SEER database. Our results showed that older age at diagnosis and higher tumor and nodal stages were associated with a worse outcome. Both CSS and OS remained poor despite the availability of targeted agents. CN was associated with improved survival in both the pre-targeted therapy and targeted therapy era. 

The results of this study are in agreement with other published studies showing poor survival and lack of benefit from targeted therapy in patients with sRCC [11,12,23-27]. In a retrospective analysis of data from the International Metastatic Renal Cell Carcinoma Database Consortium (IMDC), Kyriakopoulos et al.* *analyzed the data of 230 patients with metastatic sRCC who were diagnosed between August 2008 and January 2013 [26]. In their study, the targeted agent sunitinib was used as the first-line treatment in most patients (78%). Despite the use of targeted agents, patients had worse progression-free survival (PFS) and OS than patients with metastatic non-sarcomatoid RCC. In another study, the median OS and PFS of a cohort of 63 patients with mRCC who were treated with an antiangiogenic TKI, cytokine therapy, and chemotherapy were 3 and 10 months, respectively. Sunitinib-treated patients had a PFS of 4.4 months, compared to a PFS of two months for patients treated with other agents; 33%-57% of patients progressed in a year [27].

In contrast to the poor response rate with targeted agents, this present study has shown an improvement of CSS and OS with CN in both the pre-targeted and targeted therapy eras. While Adashek et al. demonstrated no benefit of CN in a retrospective analysis of 167 patients with sRCC [15], other studies have shown mixed results [11-14]. Keskin et al. analyzed 199 patients with metastatic sRCC who were diagnosed between 1987 and 2015 and underwent CN and systemic therapy [11]. In agreement with other studies, including our study, they reported poor OS. When the survival data were analyzed based on treatment era, an improvement in OS in the first year after diagnosis was reported for 99 patients treated with targeted therapy, but this was attenuated within two years and disappeared within 3-5 years. Aleviazakos et al. examined the data of 879 patients with sRCC in the SEER database between 2010 and 2015 [12]. Patients who had metastatic disease and underwent CN had a better probability of disease-specific survival than those patients who did not undergo CN. In a single-center study of 192 sRCC patients, there appeared to be a benefit of CN in patients with sarcomatoid differentiation associated with clear cell histology, unifocal metastasis, and node-negative disease [13]. A SEER-based analysis also showed the benefits of CN in metastatic sRCC patients, but survival was better with lower T stages (T1, T2) compared to higher T stages (T3 or higher) [14]. The results of our study are in agreement with these studies. In addition, our study directly compared the effects of CN on survival outcomes in two eras before and after the availability of targeted therapy. The results showed the beneficial effects of CN in both eras.

It is important to mention that our study included a period prior to the approval of ICI-based therapies in RCC and, therefore, does not reflect the impact of CN in the immunotherapy era in this aggressive disease. Preliminary results of ICI-based combinations in patients with sRCC are promising [17-19]. Therefore, a well-designed prospective randomized study of the effects of CN on patient outcomes in the immunotherapy era is eagerly awaited.

There are several limitations to this study. As a retrospective analysis of a population database, there are inherent limitations to this type of design. These include but are not limited to selection bias and lack of detailed and complete patient-level information. Information on the proportion of sarcomatoid components in each patient were lacking, which had prognostic implications. In addition, information on the type of targeted therapy as well as the sequence of therapy was not available. The SEER database also lacked data on additional risk factors, co-morbidities, and laboratory variables, which might influence the outcome. There was an imbalance in the numbers of patients in the two treatment eras as well as the duration of the two eras. It is possible that the diagnosis of sarcomatoid histology has increased recently due to improved recognition of sRCC. Our results should, therefore, be interpreted in this context.

## Conclusions

Based on the analysis of a SEER population-based database, this retrospective study showed that the introduction of targeted therapy was not associated with improved survival in patients with metastatic sRCC. Older age at diagnosis and higher tumor and nodal stages were associated with worse survival outcomes. CN was associated with improved survival in both the pre-targeted and targeted therapy eras in patients with mSRCC. Prospective randomized studies are required to examine the effects of CN on patient outcomes in the current immunotherapy era.
